# Efficacy and safety analyses of bevacizumab in neoadjuvant chemotherapy for ovarian cancer: a systematic review and meta-analysis

**DOI:** 10.3389/fphar.2025.1566604

**Published:** 2025-05-30

**Authors:** Yijun Wang, Yicong Wang, Lei Wang, He Yuan, Yongai Yu, Daju Liu

**Affiliations:** Department of Obstetrics and Gynecology, Dalian Municipal Central Hospital, Dalian, China

**Keywords:** ovarian neoplasms, bevacizumab, monoclonal antibodies against vascular endothelial growth factor, avastin, neoadjuvant chemotherapy

## Abstract

**Objective:**

To assess the efficacy and safety of bevacizumab in neoadjuvant chemotherapy for ovarian cancer through systematic evaluation and meta-analysis.

**Methods:**

Online databases, such as PubMed, Embase, and the Cochrane Library, were searched for relevant articles on the treatment of ovarian cancer patients with interval debulking surgery after neoadjuvant chemotherapy in combination with the bevacizumab regimen using the keywords “Ovarian Neoplasms,” “Bevacizumab,” “Monoclonal antibodies against vascular endothelial growth factor,” “Avastin,” and “Neoadjuvant Therapy.” A meta-analysis of the screened literature, which included randomized controlled trials and cohort studies, was then performed using Stata 15.0 software.

**Results:**

The meta-analysis included five eligible papers. The test group consisted of 160 patients who received paclitaxel + carboplatin + bevacizumab prior chemotherapy (TCB), whereas the control group consisted 211 patients who received paclitaxel + carboplatin (TC). The results indicate that there was no significant difference between the two groups in terms of the rate of optimal cytoreduction (RR = 1.124, 95% CI: 0.947-1.335, P = 0.182; Heterogeneity: *I* ^*2*^ = 40.3%, p = 0.152) and progression-free survival (PFS) (HR = 0.74, 95% CI: 0.48-1.14, p = 0.173; Heterogeneity: *I* ^*2*^ = 86%, p = 0.007). Neoadjuvant chemotherapy with bevacizumab did not increase the incidence of adverse events in chemotherapy (RR = 0.88, 95% CI: 0.713-1.088, p = 0.238; Heterogeneity: *I* ^*2*^ = 49.5%, p = 0.095). The rate of postoperative complications in the TCB group was comparable to that in the TC group (RR = 0.955, 95% CI:0.672-1.359, p = 0.799; Heterogeneity: *I* ^*2*^ = 6.8%, p = 0.368).

**Conclusion:**

The use of bevacizumab in neoadjuvant chemotherapy for advanced ovarian cancer was safe and feasible but did not significantly improve the satisfactory tumor reduction rate of interval debulking surgery and had no effect on the prolongation of postoperative PFS. Hence, the use of bevacizumab in preemptive chemotherapy for ovarian cancer should be carefully considered.

**Systematic Review Registration:**

https://inplasy.com/inplasy-2024-12-0065/, INPLASY2024120065.

## 1 Introduction

Ovarian cancer is one of the three most common malignant tumors affecting the female reproductive system. Given the lack of effective methods for early diagnosis, around 70% of the patients are diagnosed with advanced stage disease, and 70% of the patients experience recurrence within 2–3 years after treatment, with a 5-year survival rate of only 30%–40%, which poses a serious threat to women’s health (Armstrong et al., 2022). Although the incidence rate of ovarian cancer is lower than that of cervical and endometrial cancers, its mortality rate is more than the sum of the two, ranking first among gynecological cancers. Hence, ovarian cancer has been considered a serious threat to women’s health worldwide (Armstrong et al., 2021).

Satisfactory tumor cytoreduction followed by adjuvant platinum-based chemotherapy has been the foundation of ovarian cancer treatment. In patients with advanced ovarian cancer with FIGO stage III or IV disease, neoadjuvant chemotherapy (NACT) followed by interval debulking surgery (IDS) has been found to provide a more favorable outcome than primary debulking surgery (PDS) (Medina-Franco and Mejía-Fernández, 2018).

IDS provided similar overall survival benefit as PDS and was better tolerated. Carboplatin and paclitaxel (TC) chemotherapy remains the standard of care for NACT. However, bevacizumab, an anti-vascular endothelial growth factor monoclonal antibody, has currently seen wide usage in ovarian cancer. Moreover, evidence has shown that the combination of postoperative chemotherapy and bevacizumab improves the prognosis of patients with a large amount of ascites, those with stage IV disease, and other populations with high recurrence rates. Furthermore, studies have shown that the use of bevacizumab for follow-up maintenance therapy after the initial treatment can improve the prognosis of BRCA wild-type or unknown population who received postoperative chemotherapy combined with bevacizumab (du Bois et al., 2019). However, limited studies have been available on the use of bevacizumab for NACT in ovarian cancer, leading to inconclusive evidence regarding its risks and benefits. Therefore, the current meta-analysis aimed to analyze relevant clinical studies in order to comprehensively explore the safety of adding bevacizumab to prior chemotherapy for ovarian cancer patients, as well as its effect on IDS surgery satisfaction and patients’ survival prognosis.

## 2 Materials and methods

### 2.1 Search strategy

The INPLASY registration number that corresponds to this study is INPLASY2024120065. We conducted a comprehensive literature search of Embase, PubMed, and Cochrane Library databases and manually screened all relevant references. The search encompassed all studies included from the date at which the database was first created until the present. The search terms included “Ovarian Neoplasms,” “Bevacizumab,” “Monoclonal antibodies against vascular endothelial growth factor,” “Avastin,” and “Neoadjuvant Therapy.” No regional, racial, age, or payment restrictions were set in our search. Furthermore, literature reviews and references to original studies were scanned to avoid missing any eligible studies.

### 2.2 Selection criteria

The inclusion criteria for this meta-analysis were as follows (Armstrong et al., 2022): patients with advanced ovarian cancer undergoing neoadjuvant therapy (Armstrong et al., 2021); neoadjuvant therapy with the addition of bevacizumab (Medina-Franco and Mejía-Fernández, 2018); treatment with intermittent tumor cytoreduction (IDS) after neoadjuvant therapy (du Bois et al., 2019); study outcomes that included progression-free survival (PFS), complete tumor resection (R0/R1), adverse events (AEs) of preemptive chemotherapy, and postoperative complications; and (Rouzier et al., 2017) studies published in English.

The exclusion criteria used in this study were as follows (Armstrong et al., 2022): duplicate published studies (Armstrong et al., 2021), lack of necessary study data (Medina-Franco and Mejía-Fernández, 2018), incompatible studies, and (du Bois et al., 2019) animal experiments, cellular studies, case reports, reviews, meta-analyses, abstracts, or letters.

The Newcastle-Ottawa Scale (NOS) was used to assess the quality of non-RCTs (i.e., case–control studies or cohort studies). This scale has a total score of 9, with a score of 6 and above indicating a high-quality study and a score below 6 indicating a low-quality study. The two non-RCTs included herein obtained a score of 7 and 9, respectively (see Supplementary Table S1 for details).

For randomized controlled trials (RCTs), we referred to the Cochrane Collaboration’s set of entries for risk of bias assessment and independently assessed the risk of bias for each study. Ultimately, the total score for each study was calculated based on these criteria.

### 2.3 Data extraction and quality assessment

Two investigators independently assessed the eligibility of the included trials in two stages (Armstrong et al., 2022): title and abstract screening and (Armstrong et al., 2021) full text review. Discrepancies between the two investigators were resolved through consensus discussions and involved a third reviewer with expertise in gynecologic oncology when needed. For each trial, the investigators extracted the trial name and year of publication, first author, type of study, study methodology, sample size, patient characteristics and disease histology, PFS, number of cases with complete tumor resection (R0/R1), AEs of prior chemotherapy, and postoperative complications. In cases wherein multiple publications or reports of the same trial existed, data with the most complete information from the most recent publication were collected. Finally, two reviewers independently assessed the quality of the RCTs using the Cochrane Collaboration risk of bias tool, and differences were resolved through discussion and consultation with a third reviewer.

This study used RevMan 5.4 software to assess the risk of bias in RCTs. Among the three included studies, low and moderate risk of bias was observed regarding the generation of randomized sequences, blinding measures applied to subjects and researchers, and completeness of trial results. Overall, the quality assessment of the included RCTs indicated a moderate risk of bias (see Supplementary Figures S1, S2 and Supplementary Table S2 for details).

### 2.4 Statistical analysis

Meta-analysis was performed using Stata 15.0 software. First, heterogeneity was assessed using Cochran’s Q test (α = 0.10) and I^2^ statistics. We considered I^2^ >50% or a significant Q test (p < 0.10) as indicative of substantial heterogeneity. A fixed-effects model (Mantel-Haenszel method) was used if low heterogeneity (I^2^ ≤ 50%) was observed; otherwise, a random-effects model (DerSimonian-Laird method) was applied. Satisfactory tumor cytoreduction rate, prior chemotherapy AEs, and surgical complications were assessed using relative risk (RR) values and 95% CI, whereas PFS was assessed using hazard ratios (HRs). Publication bias was assessed using Egger’s test on Stata 15.0 and funnel plots. Moreover, sensitivity analysis was performed to assess the impact of study quality on the overall conclusions. Finally, graph effect sizes and confidence intervals were determined based on the results of the meta-analysis using methods such as forest plots. Other statistical methods were also used to interpret and analyze inter-study heterogeneity as needed. All results were presented in the form of tables and graphs.

## 3 Results

### 3.1 Eligible studies

**FIGURE 1:**
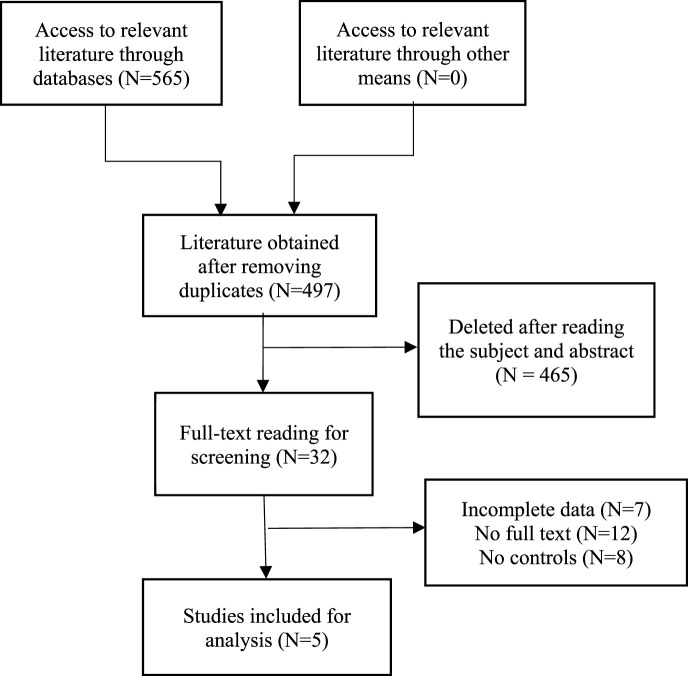
Flowchart for this meta-analysis.

After a comprehensive search of major databases, a total of 565 relevant articles were retrieved. After removing duplicate articles, a total of 497 articles remained. Subsequently, we conducted a detailed review of the titles and abstracts of the identified studies based on set inclusion and exclusion criteria, resulting in the exclusion of 465 articles. After carefully reviewing the full text, five articles were finally included for analysis, enrolling a total of 371 patients with stage III or IV ovarian cancer, among whom 160 received prior chemotherapy with the TCB regimen and another 211 received chemotherapy with the TC regimen. Study types included three RCTs and two cohort studies. Table 1 summarizes the characteristics of the included studies, listing their basic information and case characteristics. A flow chart for the screening of the studies is detailed in Figure 1.

**TABLE 1:** Characteristics of studies included in this meta-analysis.

Author	Date of publication	Type of study	Number of patients (TCB/TC)	Age (TCB/TC)	Installments	Number of R0 (TCB/TC)	Number of R1 (TCB/TC)	Surgical time (TCB/TC)	Number of adverse events of upfront chemotherapy (TCB/TC)	Number of perioperative complications (TCB/TC)	PFS (TCB/TC)
Rouzier et al. (2017)	2017	RCT	58/37	63 (33–87)/63 (39–79)	IIIC/IV	34/19	3/2	NR	34/25	11/8	NR/NR
Kusunoki et al. (2018)	2018	Cohort	11/13	59 (30–67)/57 (38–68)	IIIC/IV	9/9	2/NR	median:254min/314min	9/12	3/4	NR/NR
Garcia Garcia et al. (2019)	2019	RCT	35/33	63 (33–78)/57 (36–82)	III/IV	9/8	11/6	271min (35–560)/260min (147–570)	10/20	5/2	HR (95%CI) = 1.13 (0.66–1.93), p = 0.664
Park et al. (2020)	2020	Cohort	16/88	56 (39–78)/58 (39–77)	IIIC–IV	9/42		NR	4/10	8/30	HR (95%CI) = 0.32 (0.22–0.99), p = 0.048
Yin et al. (2022)	2022	RCT	40/40	median: 55	IIIC/IV	33/25		NR	17/18	10/15	NR

Author	Stage IIIC (TCB/TC)	Stage IV (TCB/TC)	Plasmacytoma (TCB/TC)	Endometrioid carcinoma (TCB/TC)	Mucinous carcinoma (TCB/TC)	Clear cell carcinoma (TCB/TC)	High differentiation (TCB/TC)	CA125 at diagnosis (TCB/TC)	CA125 after preemptive chemotherapy (TCB/TC)
Rouzier et al. (2017)	43/24	15/13	54/36	1/0	NR	NR	53/31	1008 (128–37537)/1281 (44–54152)	NR
Garcia Garcia et al. (2019)	23/22	12/11	27/26	1/2	NR	NR	8/9	NR	NR
Yin et al. (2022)	32/33	8/7	28/25	7/8	2/3	3/4	35/32	NR	NR
Kusunoki et al. (2018)	7/8	4/5	NR	NR	NR	NR	16/80	645 (245–247800)/1606 (498–18770)	17 (11–642)/24 (8–198)
Park et al. (2020)	3/37	13/51	NR	NR	NR	NR	NR	4600.20 ± 4823.63/2727.73 ± 3848.45	136.77 ± 194.79/281.06 ± 1663.09

Abbreviations: TCB, paclitaxel + carboplatin + bevacizumab group; TC, paclitaxel + carboplatin group; R0, tumor resection with no residuals to the naked eye; R1, residual tumors <1 cm in diameter; NR, no data available; PFS, progression-free survival; AEs, adverse events occurring during treatment.

### 3.2 Efficacy evaluation

A total of 371 patients with advanced ovarian cancer were included in the study, among whom 232 and 139 had stage IIIC and stage IV disease. Plasma carcinoma was the main pathologic type, with a few other pathologic types such as endometrioid carcinoma, mucinous carcinoma, and clear cell carcinoma. The test group consisted of 160 patients who received paclitaxel + carboplatin + bevacizumab (TCB) totaling, whereas the control group consisted of 211 who received paclitaxel + carboplatin (TC) (Table 1).

**FIGURE 2:**
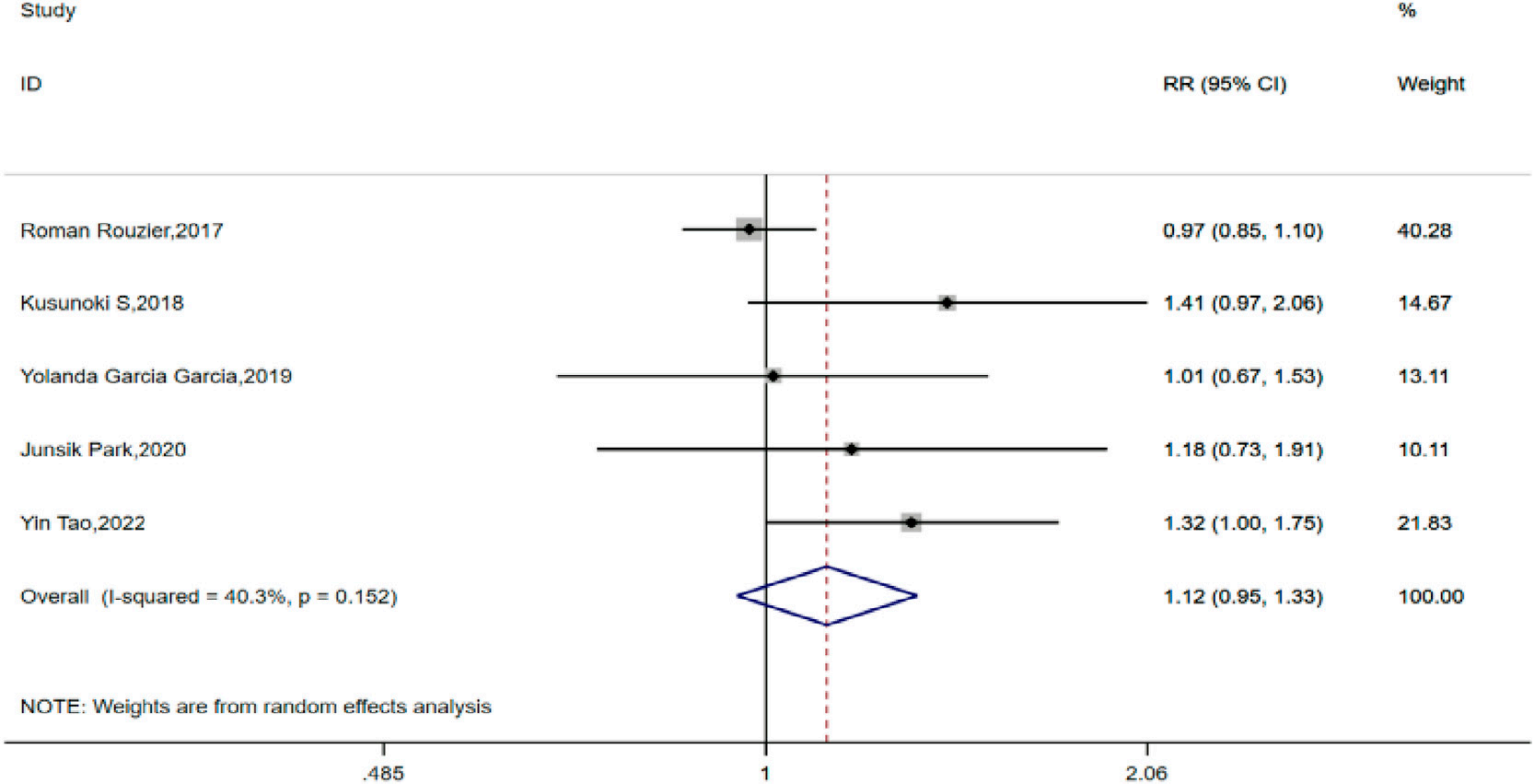
Forest plots for satisfaction rate for complete tumor resection.

All five of the included studies compared the satisfactory tumor cell reduction (R0 + R1) rates between the TCB and TC groups for IDS. After testing for heterogeneity, a fixed-effects model was used for analysis (H:I^2^ = 40.3%, P = 0.152), with all five studies showing a pooled RR value of 1.124, with a 95% CI of 0.947–1.335 (P = 0.182), indicating no statistically significant difference in the rate of satisfactory tumor cell reduction after interval debulking surgery between patients who received preemptive chemotherapy with or without bevacizumab for advanced ovarian cancer (Figure 2).

**FIGURE 3:**
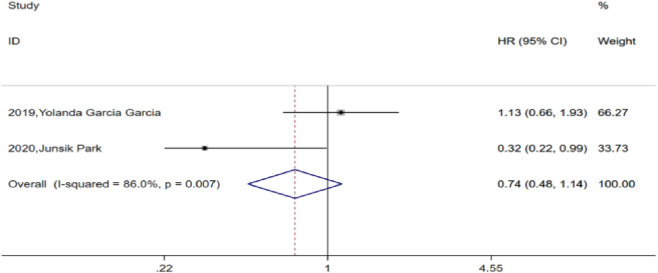
Forest plots for progression-free survival (PFS) between TC and TCB regimens.

Among the included studies, two compared PFS between the TCB and TC groups. After heterogeneity testing (H:I^2^ = 86%, p = 0.007), both studies showed I^2^ = 86% > 50%, suggesting heterogeneity between the selected studies. Further examination of the funnel plot suggested that the heterogeneity of the included studies was within an acceptable range and were therefore retained for analysis using a random-effects model. The pooled HR for the two studies was 0.74, with a 95% CI of 0.48–1.14 (p = 0.173), which was not statistically significant. This finding suggests that combined bevacizumab for prior chemotherapy in advanced ovarian cancer did not significantly improve PFS compared to TC alone (Figure 3).

Moreover, two of the included studies compared changes in CA125 at diagnosis and after preemptive chemotherapy. According to the Gynecologic Cancer Intergroup definition of a CA125 response in preemptive chemotherapy (i.e., a 50% or more decrease in CA125 levels lasting for at least 28 days), chemotherapy in both the TCB and TC groups was effective.

### 3.3 Safety assessment

**FIGURE 4:**
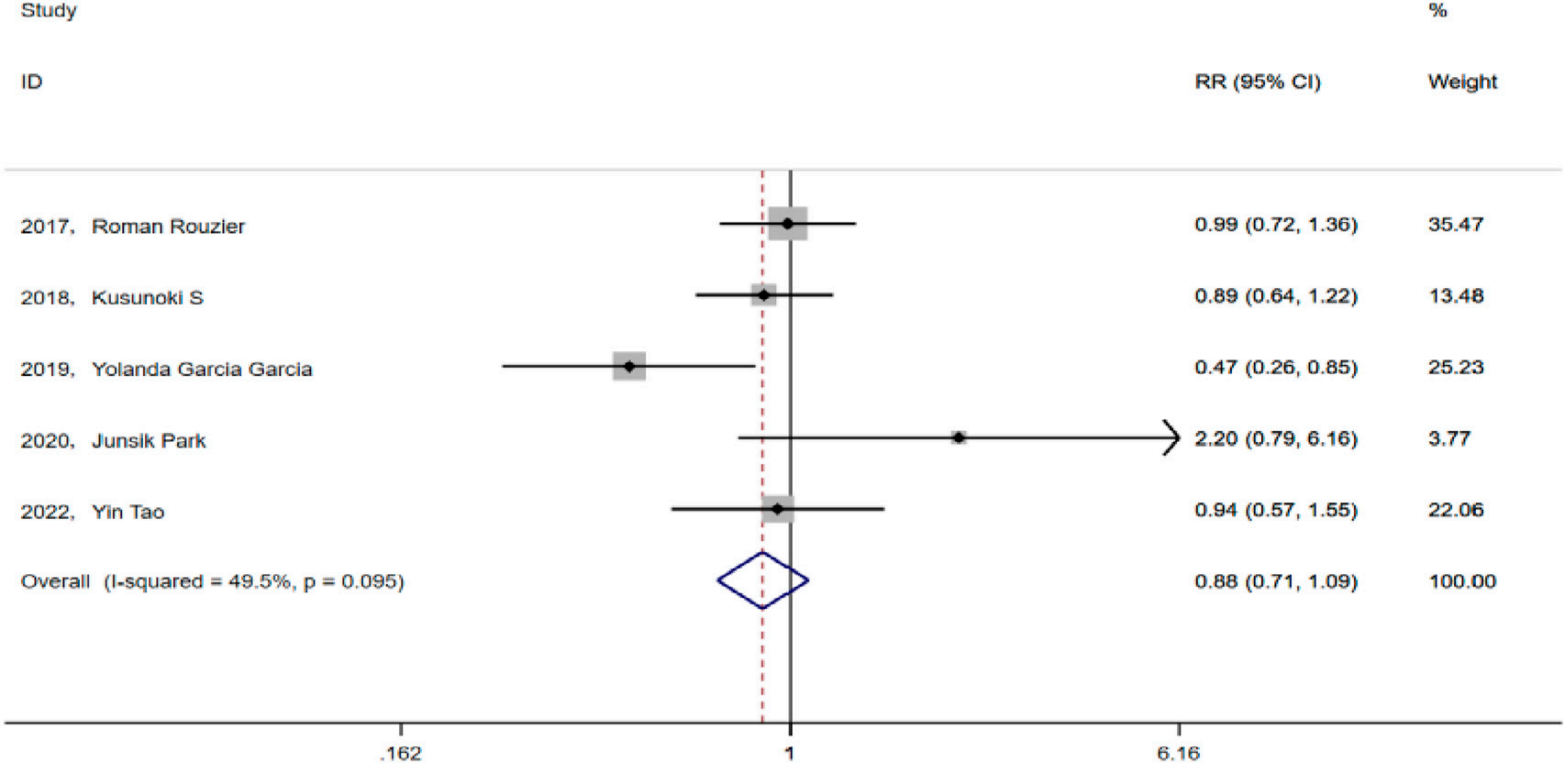
Forest plots for chemotherapy-related adverse events between TC and TCB regimens.

All five studies included herein analyzed AEs in prior chemotherapy, mainly including anemia, fever, thrombocytopenia, malaise, and gastrointestinal reactions. After heterogeneity testing, we analyzed the results using a fixed effect model (I^2^ = 49.5% < 50%, p = 0.095), subsequently revealing that the five studies had a pooled RR value of 0.88 and a 95% CI of 0.713–1.088 (p = 0.238). This finding suggests that the addition of bevacizumab to preemptive chemotherapy of advanced ovarian cancer does not increase the incidence of side effects during chemotherapy (Figure 4).

**FIGURE 5:**
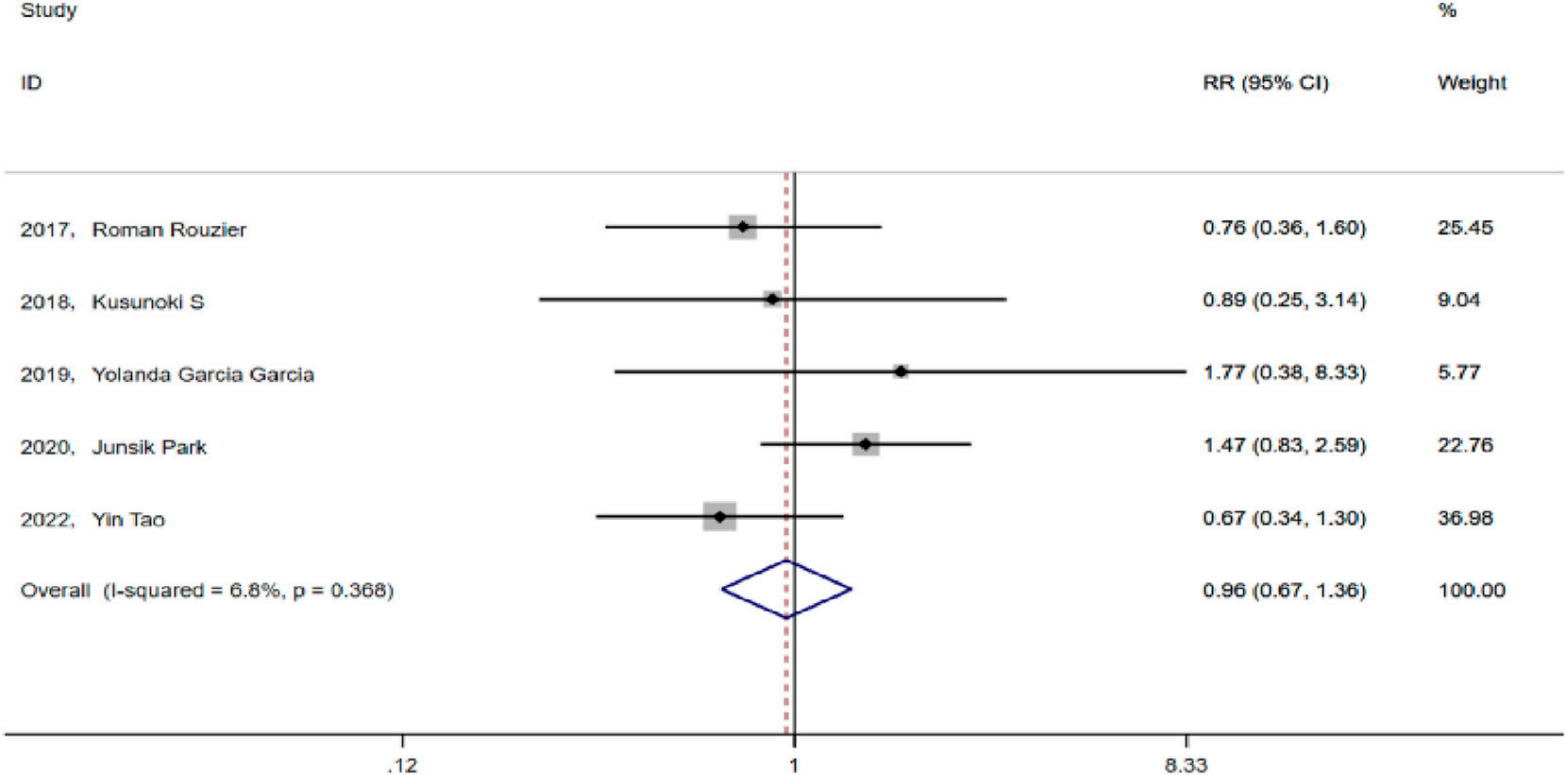
Forest plots for perioperative complication rates between TC and TCB regimens.

Intraoperative and postoperative complications in the TCB and TC groups mainly included bleeding, infection, poor incision healing, and gastrointestinal complications, among others. After heterogeneity testing, I^2^ = 6.8% < 50% (p = 0.368), suggesting no significant heterogeneity among the selected studies. Hence, we selected the fixed effect model for analysis. The pooled RR value of the five studies was 0.955, with a 95% CI of 0.672–1.359, but was not statistically significant (p = 0.799). This finding suggests that in advanced ovarian cancer, prior chemotherapy with bevacizumab did not increase the incidence of perioperative complications (Figure 5).

### 3.4 Publication bias

We then examined whether publication bias existed in the included study by creating funnel plots. Notably, the results of the funnel plots showed that complete tumor resection rate and PFS were symmetrical and that no publication bias existed (Supplementary Figures S3, S4). Moreover, the generated funnel plots revealed AEs in prior chemotherapy were found to be biased in one article. However, the generated sensitivity plots showed that the biased articles were within the acceptable range and were therefore retained (Supplementary Figures S5, S6). The funnel plots for perioperative complications showed no bias (Supplementary Figure S7). Within the acceptable range, so the inclusion was retained (Supplementary Figures S5, S6).

## 4 Discussion

To evaluate the efficacy and safety of bevacizumab in prior chemotherapy for ovarian cancer, the current study analyzed five studies involving 371 patients. Accordingly, our findings showed no significant differences in the rate of satisfactory tumor cell reduction (RR = 1.124, 95% CI: 0.947–1.335, P = 0.182; H:I^2^ = 40.3%, P = 0.152) and PFS (HR = 0.74, 95% CI. 0.48–1.14, P = 0.173; H:I^2^ = 86%, P = 0.007) between the TCB and TC groups. Combining preemptive chemotherapy with bevacizumab did not increase the incidence of AEs during chemotherapy (RR = 0.88, 95% CI: 0.713–1.088, P = 0.238; H:I^2^ = 49.5%, P = 0.095), and the rate of postoperative complications in the TCB group was comparable to that in the TC group (RR = 0.955, 95% CI: 0.672–1.359. P = 0.799; H:I^2^ = 6.8%, P = 0.368). Therefore, our findings suggest that combining bevacizumab with prior chemotherapy for advanced ovarian cancer was safe and feasible but does not significantly improve the rate of satisfactory tumor reduction with IDS or prolong postoperative PFS. Hence, the use of bevacizumab in preemptive chemotherapy for ovarian cancer should be carefully considered.

At present, NACT for ovarian cancer remains controversial (Wright et al., 2016; Rauh-Hain et al., 2012; Gadducci et al., 2017; Leary et al., 2016; van der Burg et al., 1995). The 2024 NCCN guidelines (Armstrong et al., 2024) state that IDS after NACT may be considered for advanced ovarian cancers evaluated by gynecologic oncologists and for which initial surgery failed to achieve satisfactory reduction. Although IDS after NACT had been found to increase R0 resection rates and reduce surgical complications, PFS and OS did not significantly differ from those of PDS (Vergote et al., 2018; van Meurs et al., 2013). The preferred regimen for prior chemotherapy is the same as that for stage II–IV ovarian cancer, and the NCCN guidelines recommend 3 weeks of TC with or without bevacizumab (Armstrong et al., 2024). However, limited studies have been available on the use of bevacizumab for NACT in ovarian cancer, with inconclusive evidence available regarding its risks and benefits.

Bevacizumab, the first anti-angiogenesis targeted drug, is a humanized IgG1-type monoclonal antibody targeting vascular endothelial growth factor. With the accumulation of research-based medical evidence, bevacizumab has received international approval for the treatment of colorectal cancer, lung cancer, breast cancer, hepatocellular carcinoma, ovarian cancer, and cervical cancer. It has also been approved in China for the treatment of metastatic colorectal cancer and advanced, metastatic, or recurrent non-small cell lung cancer, hepatocellular carcinoma, epithelial ovarian cancer (including fallopian tube or primary peritoneal cancer), and cervical cancer. These findings suggest that the use of bevacizumab in ovarian cancer is gradually becoming more widespread and that the combination of chemotherapy and bevacizumab for populations at high risk for recurrence, such as patients with large amounts of ascites and those with stage IV disease, can improve prognosis (Ferriss et al., 2015; Tewari et al., 2019; Burger et al., 2018). For BRCA wild-type or unknown populations who had received initial chemotherapy combined with bevacizumab, subsequent maintenance therapy with bevacizumab after initial treatment had been found to be helpful for improving prognosis (Burger et al., 2011; Perren et al., 2011; Oza et al., 2015).

Unfortunately, only a handful of studies have been published on the use of bevacizumab for NACT in ovarian cancer, with its risks and benefits being unclear and its efficacy still remaining controversial. The ANTHALYA trial (Rouzier et al., 2017) enrolled 95 patients with FIGO stage IIIC/IV ovarian cancer randomized to receive four cycles of neoadjuvant TC ± 3 cycles of bevacizumab 15 mg/kg (TCB) in a randomized group (2:1), followed by IDS. Thereafter, IDS was performed, with the main objective of evaluating the complete resection ratios (CRR) of IDS in the TCB group. Notably, the study found a CRR of 58.6% and 51.4% in the TCB and TC groups, respectively, which the former being significantly higher than the latter. Komiyama et al. (Komiyama et al., 2018) studied 23 patients with advanced ovarian cancer who could not undergo complete resection during exploratory surgery by cesarean section and were started on TCB chemotherapy followed by IDS 12–14 days after exploratory surgery. Their results showed that one patient achieved complete response, whereas 19 patients achieved partial response, with a response rate of 86.9% (95% CI: 66.4%–97.2%), suggesting that NACT + bevacizumab followed by IDS was an acceptable therapeutic strategy in terms of safety and surgical outcomes for patients with advanced ovarian cancer who undergo complete resection during exploratory surgery. The MITO-16A-MaNGO OV2A study (Daniele et al., 2017) was a non-randomized controlled study evaluating bevacizumab in combination with paclitaxel and carboplatin for stage IV ovarian cancer. Subgroup analyses in the mentioned study showed that 86.5% of patients who underwent IDS had residual lesions ≤1 cm after surgery. Moreover, the mentioned study found that TCB was an acceptable treatment strategy for advanced ovarian cancer in combination with chemotherapy. However, whether TCB can improve the rate of complete resection of IDS still remains unclear. The GEICO 1205 study (Garcia Garcia et al., 2019) compared chemotherapy alone with chemotherapy combined with bevacizumab for neoadjuvant treatment of patients with advanced ovarian cancer. Both groups of patients received chemotherapy combined with bevacizumab and sequential application of bevacizumab maintenance therapy after surgery. Notably, their results showed that patients with advanced ovarian cancer treated with NACT combined with bevacizumab showed no benefit in terms of median PFS time (20.1 months:20.4 months; P = 0.66) but that the feasibility of IDS was higher in the NACT combined with bevacizumab group than in the chemotherapy alone group (67%:89%, P = 0.029). Zhang et al. (2023) evaluated the modality, compliance, efficacy, and safety of bevacizumab in the treatment of Chinese patients with ovarian cancer. Notably, their findings revealed that ten patients were assessed as stable disease (SD) via imaging after completing three cycles of preemptive chemotherapy; 18 patients showed a <50% decrease in CA125 after completing one cycle of chemotherapy; 38 out of 43 (88.4%) patients who underwent IDS reached optimal reduction (residual tumor <1 cm); and 24 patients (55.8%) had no residual tumor after IDS. The investigators concluded that bevacizumab was effective in the treatment of ovarian cancer and that the addition of bevacizumab to NACT is feasible. The results of GEICO 1205 (Garcia Garcia et al., 2019) found that the median PFS for the TCB group and TC group were 20.4 and 20.1 months, respectively, whereas the 1-year PFS was 88% and 68% in the bevacizumab group and chemotherapy-only group. These findings demonstrate that the addition of three to four cycles of preoperative bevacizumab in unresectable NACT did not significantly improve PFS but improved surgical maneuverability without increasing toxicity. Therefore, the investigators supported the early use of bevacizumab in selected high risk patients with unresectable ovarian cancer. In the current study, we found that the TCB had higher response rates than did the TC group but that no significant differences in the complete resection rate of IDS (RR = 1.124, 95% CI:0.947–1.335, P = 0.182; H:I^2^ = 40.3%, P = 0.152) and PFS (HR = 0.74, 95% CI:0.48–1.14, P = 0.173; H:I^2^ = 86%, P = 0.007) were observed between the two groups. In conclusion, we found that similar to previous studies, the addition of bevacizumab to preemptive chemotherapy improved the chances of surgery and reduced surgical complications of PDS in critically ill patients but did not improve the rate of satisfactory tumor cell attenuation and did not promoted any significant improvement in prognosis when compared to the traditional TC regimen. As such, large prospective RCTs are warranted needed.

According to early studies, the most common toxic reactions to bevacizumab include hypertension and proteinuria, as well as mucosal bleeding, thrombosis, and gastrointestinal toxicities such as perforation, bleeding, and fistula formation (Wang et al., 2023). Although routine doses of bevacizumab therapy are tolerated by most patients and are safe and manageable, toxic reactions occurring in patients receiving bevacizumab therapy, which may include: pain (≥grade 2), neutropenia (≥grade 4), febrile neutropenia, thrombocytopenia, and hemorrhage (≥grade 2; various types), hypertension (≥grade 2), thromboembolism (≥grade 3; various types), gastrointestinal events (perforations, abscesses, and fistulas), reversible posterior white matter encephalopathy syndrome, renal injury and proteinuria (≥grade 3), and wound rupture, may require physician intervention as they often result in interruption of therapy. In GOG-0218 and ICON7, bleeding, hypertension, proteinuria, thromboembolic events (≥grade 3), gastrointestinal perforation (≥grade 3), and wound healing complications were found to be common in the bevacizumab group (Burger et al., 2011; Perren et al., 2011). Therefore, careful observation and management of common adverse effects of bevacizumab are needed. Elderly patients (≥70 years old) and/or those with co-morbidities are prone to chemotherapy side effects and may not tolerate certain combination chemotherapy regimens, leading to the discontinuation of the regimen before completion (Hilpert et al., 2007; Hershman et al., 2016; Selle et al., 2018; Fairfield et al., 2011; Falandry et al., 2019). For example, studies have shown that patients 70 years of age or older who were treated with paclitaxel + carboplatin may be at higher risk for febrile neutropenia, anemia, diarrhea, weakness, thromboembolic events, or hypertension (Hilpert et al., 2007). Data from GOG-0218 and ICON7 showed that most toxicities occurred during the chemotherapy phase of treatment, although some AEs of concern, including hypertension, severe pain, proteinuria, and thromboembolism, continued to occur during the maintenance phase of bevacizumab. Exploratory analyses have attempted to identify factors that may be associated with an increased risk for bevacizumab-related AEs (Burger et al., 2014; Duska et al., 2015).

The 2018 ESMO-EGSO guidelines for ovarian cancer (Ray-Coquard et al., 2018) stated that bevacizumab can be safely administered before and after IDS; however, the interval between surgery and administration should be at least 4–6 weeks. Wang et al. (2023) found that hypertension was the most common adverse event associated with bevacizumab and that 20 patients with epithelial ovarian cancer (25.3%) had new-onset hypertension or worsening of pre-existing hypertension after bevacizumab exposure, with a dose-dependent trend in bevacizumab-associated blood pressure changes. Among all 46 AEs, only 8 (8.9%) were categorized as grade ≥3 toxicity; the incidence of bevacizumab-related bleeding was 3.8%; 15.2% of patients suffered new-onset proteinuria or worsening of pre-existing proteinuria after bevacizumab exposure; and two patients (2.5%) suffered cerebral infarction after bevacizumab exposure. Thus, the presence of bevacizumab-associated blood pressure changes showed a dose-dependent trend, although the cumulative bevacizumab dose was not significantly correlated with other AEs. Bevacizumab should therefore be used with caution among patients with potential risk factors for developing bevacizumab-associated gastrointestinal perforation (GIP). Tao et al. used a TCB regimen with intraperitoneal instillation of bevacizumab in NACT for advanced ovarian cancer and found that it promoted reduced intraoperative blood loss, decreased operative time, increased rate of patient satisfaction with surgery, and reduced incidence of postoperative wound infections, hypoproteinemia, abdominal distension, and fever when compared to the TB regimen. The use of bevacizumab for the treatment of advanced ovarian cancer was associated with a decreased risk for the development of bevacizumab-associated GIP. Yolanda Garcia Garcia et al. (2019) found that the incidence of grade ≥3 AEs was significantly lower in the TCB group of prior chemotherapy than in the TC group (54% and 79%; p = 0.033) and that the addition of bevacizumab to NACT did not increase the incidence of grade ≥3 AEs. In the current study, we found that combining bevacizumab with preemptive chemotherapy did not increase the incidence of AEs during chemotherapy and that the perioperative complication rate in the TCB group was comparable to that in the TC group, which is consistent with the findings reported in the literature.

This study has the following limitations (Armstrong et al., 2022): only a few relevant research articles were available and databases like trial registries (e.g., ClinicalTrials.gov) should be searched for additional records in future, particularly unpublished or ongoing trials (Armstrong et al., 2021); several included studies had missing data that made subgroup analyses difficult like analysis of bevacizumab-related adverse events (Medina-Franco and Mejía-Fernández, 2018); some of the findings originated from population-based cohort registry studies, which have poor level of evidence when compared to RCTs; and (du Bois et al., 2019) overall survival data, which represents the ultimate goal of tumor therapy, was obtained from only one study, which made statistical analysis difficult.

## 5 Conclusion

The current meta-analysis has been the first to assess the efficacy and safety of adding bevacizumab to prior chemotherapy for ovarian cancer. Our results showed that bevacizumab could be safely added to neoadjuvant chemotherapy for patients with ovarian cancer without increasing the incidence of AEs associated with neoadjuvant chemotherapy and surgery. However, it did not significantly improve the rate of satisfactory tumor reduction with IDS or prolong the patients’ postoperative PFS. As such, careful consideration is need for when using bevacizumab in preemptive chemotherapy for ovarian cancer. However, this study also has some limitations that warrant discussion. Accordingly, the number of articles included herein was quite small, the research direction of the articles varied considerably, the data collected were insufficient, and some key findings originated from large population-based cohort registry studies with poor level of evidence compared to RCTs. We believe that future large-scale prospective studies would be able to address the limitations of the current study.

## Glossary

Ovarian neoplasms: tumors or cancer of the ovary. these neoplasms can be benign or malignant. they are classified according to the tissue of origin, such as the surface epithelium, the stromal endocrine cells, and the totipotent germ cells. bevacizumab: an anti-VEGF humanized murine monoclonal antibody. It inhibits VEGF receptors and helps to prevent pathologic angiogenesis. neoadjuvant therapy: preliminary cancer therapy (chemotherapy, radiation therapy, hormone/endocrine therapy, immunotherapy, hyperthermia, induced etc.) that is given before the main therapy.

## Data Availability

The original contributions presented in the study are included in the article/Supplementary Material, further inquiries can be directed to the corresponding authors.
