# Supplementary material for: Efficacy and safety analyses of bevacizumab in neoadjuvant chemotherapy for ovarian cancer: a systematic review and meta-analysis
Source: Front Pharmacol. 2025 May 30;16:1566604. doi: 10.3389/fphar.2025.1566604 (PMC12162499; doi:10.3389/fphar.2025.1566604)
Supplement: Supplementary file 1 [file DataSheet1.docx]

**Supplementary materials**

Supplementary Figure 1


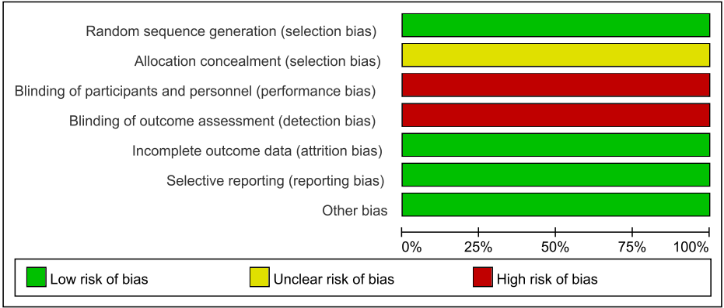


Supplementary Figure 2


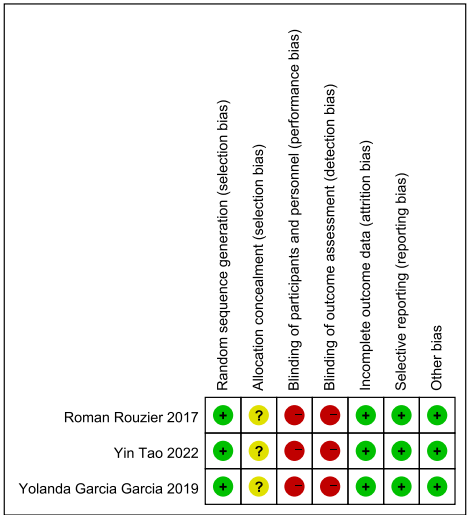


Supplementary Table 1

| Inclusion of studies | Selection | Comparability | Outcome | total |
| --- | --- | --- | --- | --- |
| Junsik Park 2020 | ☆☆☆☆ | ☆☆ | ☆☆☆ | 9 stars |
| Kusunoki S 2018 | ☆☆☆☆ | ☆☆ | ☆ | 7 stars |

Supplementary Figure 3 ：the satisfactory tumor cell reduction (R0 + R1) rates


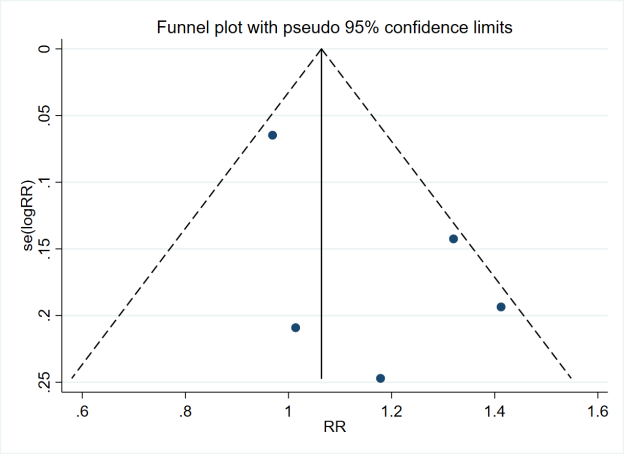


Supplementary Figure 4 ：PFS


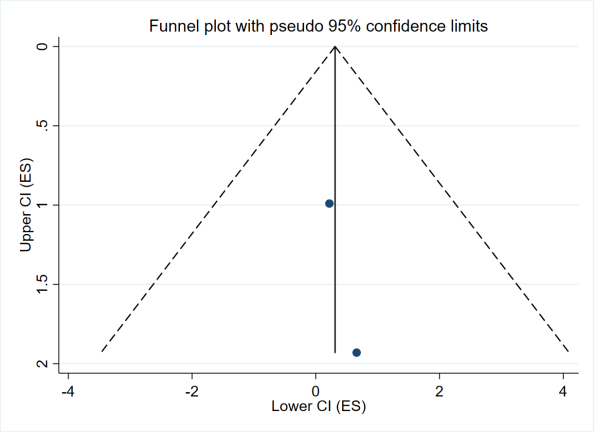


Supplementary Figure 5:the incidence of side effects during chemotherapy


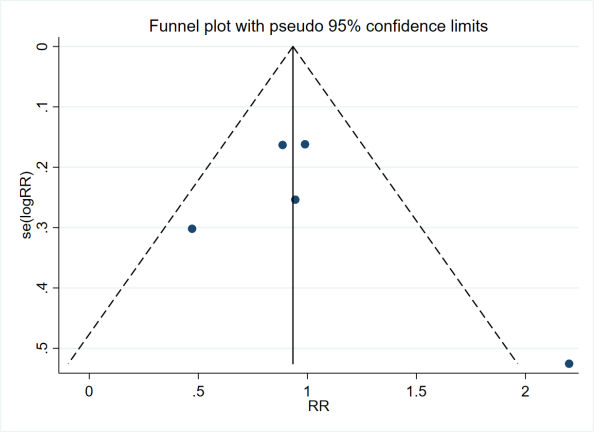


Supplementary Figure 6:the incidence of side effects during chemotherapy


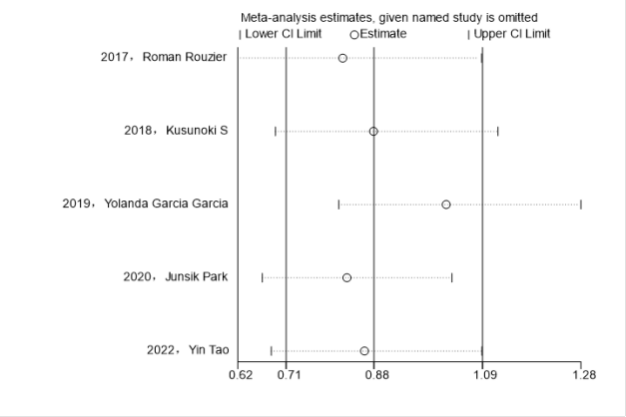


Supplementary Figure 7 ：the incidence of perioperative complications


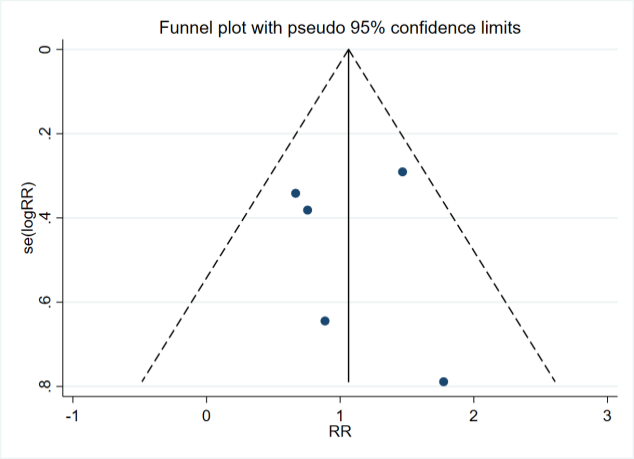


| Inclusion of studies | | randomization | Assignment hiding | blinding | Withdrawal and exit | Jadad Scale Total Score |
| --- | --- | --- | --- | --- | --- | --- |
| Roman Rouzier | 2017 | 2 | 1 | 0 | 1 | 4 |
| Yolanda Garcia Garcia | 2019 | 2 | 1 | 0 | 1 | 4 |
| Yin Tao | 2022 | 2 | 1 | 0 | 1 | 4 |

Supplementary Table 2
